# Comparative Venom Proteomics of Iranian, *Macrovipera lebetina cernovi*, and Cypriot, *Macrovipera lebetina lebetina*, Giant Vipers

**DOI:** 10.3390/toxins14100716

**Published:** 2022-10-20

**Authors:** Parviz Ghezellou, Melissa Dillenberger, Seyed Mahdi Kazemi, Daniel Jestrzemski, Bernhard Hellmann, Bernhard Spengler

**Affiliations:** 1 Institute of Inorganic and Analytical Chemistry, Justus Liebig University Giessen, 35392 Giessen, Germany; 2 Biochemistry and Molecular Biology, Interdisciplinary Research Center, Justus Liebig University Giessen, 35392 Giessen, Germany; 3 Zagros Herpetological Institute, Qom 3715688415, Iran; 4 Institute of Occupational Medicine, Social Medicine and Environmental Medicine, Goethe University, 60590 Frankfurt am Main, Germany; 5 Faculty of Forest Sciences and Forest Ecology, Department of Forest Zoology and Forest Conservation, University of Göttingen, Büsgenweg 3, 37077 Göttingen, Germany; 6 Institute of Nutritional Science, Department of Nutrition in Prevention & Therapy, Justus Liebig University Giessen, 35392 Giessen, Germany

**Keywords:** *Macrovipera lebetina cernovi*, *Macrovipera lebetina lebetina*, venom, proteomics, mass spectrometry, Iran, Cyprus

## Abstract

Envenoming by *Macrovipera lebetina* subspecies causes severe life-threatening difficulties for people living in North Africa and the Middle East. To better understand the pathophysiology of envenoming and improve patient management, knowledge about the venom components of the subspecies is essential. Here, the venom proteomes of *Macrovipera lebetina lebetina* from Cyprus and *Macrovipera lebetina cernovi* from Iran were characterized using RP-HPLC separation of the crude venom proteins, SDS-PAGE of fractionated proteins, and LC-MS/MS of peptides obtained from in-gel tryptic digestion of protein bands. Moreover, we also used high-resolution shot-gun proteomics to gain more reliable identification, where the whole venom proteomes were subjected directly to in-solution digestion before LC-HR-MS/MS. The data revealed that both venoms consisted of at least 18 protein families, of which snake venom Zn^2+^-dependent metalloprotease (SVMP), serine protease, disintegrin, phospholipase A2, C-type lectin-like, and L-amino acid oxidase, together accounted for more than 80% of the venoms’ protein contents. Although the two viper venoms shared mostly similar protein classes, the relative occurrences of these toxins were different in each snake subspecies. For instance, P-I class of SVMP toxins were found to be more abundant than P-III class in the venoms of *M. l. cernovi* compared to *M. l. lebetina*, which gives hints at a more potent myonecrotic effect and minor systemic hemorrhage following envenoming by *M. l. cernovi* than *M. l. lebetina*. Moreover, single-shot proteomics also revealed many proteins with low abundance (<1%) within the venoms, such as aminopeptidase, hyaluronidase, glutaminyl-peptide cyclotransferase, cystatin, phospholipase B, and vascular endothelial growth factor. Our study extends the in-depth understanding of the venom complexity of *M. lebetina* subspecies, particularly regarding toxin families associated with envenoming pathogenesis and those hard-detected protein classes expressed in trace amounts.

## 1. Introduction

Venomous snakes, belonging to the family Viperidae (true vipers and pit vipers), include the most medically relevant species which cause the majority of snakebite envenomings and fatalities in humans and their domestic animals [1]. Venoms produced by these snakes contain potent toxins that play a crucial role in the pathophysiology of victims of envenoming. The clinically most relevant effect of viperid bites is usually complex, involving local tissue damage such as edema and blistering, dermo- and myonecrosis, and systemic alterations like hemorrhage, coagulopathy, cardiovascular disturbances, and renal damage [1].

Members of the family Viperidae are distributed across Europe, Africa, and Asia, including the genera *Cerastes, Daboia*, *Eristicophis*, *Macrovipera*, *Montivipera*, *Pseudocerastes*, and *Vipera* [2]. The genus, *Macrovipera* or the giant Palearctic vipers, comprise three species named *M. schweizeri* (found on the Greek islands of the Cyclades Archipelago), *M. razii* (native to Southern and Central Iran), and *M. lebetina* (widely distributed from Eastern Europe to Central Asia and the Middle East) [3]. *M. lebetina* is further divided into the five subspecies *M. l. cernovi* (Iran, Afghanistan, Pakistan, India, Turkmenistan, and Uzbekistan), *M. l. obtusa* (Turkey, Russia, Syria, Iran, Iraq, Armenia, Azerbaijan, Russia, Georgia, Lebanon, Kazakhstan, and Jordan), *M. l. turanica* (Turkmenistan, Tajikistan, and Uzbekistan), *M. l. transmediterranea* (Tunisia and Algeria), and *M. l. lebetina* (Cyprus) [4,5]. They are responsible for the significant number of envenoming annually in Africa and Western Asia. Since they can deliver significant amounts of venom, they have also gained a more dangerous reputation. Envenoming by *M. lebetina* subspecies can cause severe life-threatening complications, e.g., haemorrhage, inflammation, dermo- or myonecrosis, merged with long-term musculoskeletal disabilities [6,7,8]. Furthermore, venoms of all subspecies have shown extremely potent procoagulant activity on human plasma by activating Factor X and V enzymes of the coagulation cascade [9,10]. However, information is insufficient regarding their detailed venom compositions and clinical manifestations after envenoming caused by most of these widely distributed giant vipers.

So far, proteomic studies have provided considerable information on the proteome contents and relative abundances of toxin families in a few *M. lebetina* subspecies, including Tunisian *M. l. transmediterranea* [11], Armenian *M. l. obtusa* [12], and Russian *M. l. obtusa* [13]. The results demonstrated apparent quantitative and qualitative differences between the venom compositions of these subspecies. However, toxin families belonging to snake venom Zn^2+^-dependent metalloprotease (SVMP), snake venom serine protease (SVSP), disintegrin (DISI), phospholipase A2 (PLA2), and C-type lectin-like (CTL-like) protein comprised the most dominant components of their venoms. Such potent biologically active toxins in their venoms can explain some of the leading local and systemic manifestations (e.g., edema, cutaneous necrosis, bleeding, and coagulopathies) following *M. lebetina* envenoming. Hence, much is still unknown about the venom proteome of *M. lebetina* subspecies.

Currently, the administration of an appropriate antivenom remains the only effective treatment for snakebite envenoming. However, antivenom efficacy is limited to snake species whose venoms were used in manufacture due to inter- and intraspecific variations of venom components [14]. These venom variations may result from, e.g., evolutionary history, climatic factors, ontogeny, or adaptation toward different prey, among others [15]. Therefore, most available antivenoms are limited to distinct geographical regions and may not effectively neutralize the venom toxins of other species and subspecies of snakes in different locales for therapeutic purposes. For this reason, knowledge of snake venom composition and related variation among conspecific populations can provide important information for predicting the likely efficacy of an existing antivenom, along with influencing the design of more effective immunizing mixtures for future antivenom production [16].

**Figure 1:**
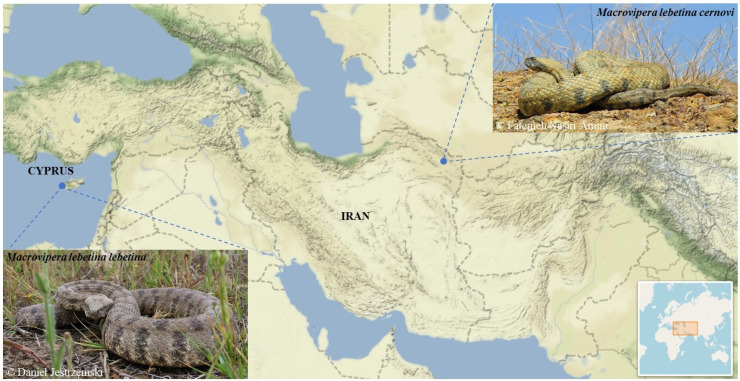
Geographical map of the collection sites. Two female specimens of *M. l. lebetina* were used for milking, which were the F2 generation of blunt-nosed vipers originally caught nearby the cities of Paphos and Polis (Paphos district, Republic of Cyprus). In comparison, two female specimens of *M. l. cernovi* were collected and milked from Khorasan Razavi, Iran. Map provided by using the “leafletR” package [26].

In this study, we performed comprehensive venom proteomics of two medically important *M. lebetina* subspecies, *M. l. lebetina* from Cyprus and *M. l. cernovi* from Iran (Figure 1), which have not yet been thoroughly investigated. The subspecies *M. l. lebetina* or Cypriot blunt-nosed viper is exclusively limited to Cyprus and is found all over the country mostly on agricultural fields and rocky slopes [17]. Adult blunt-nosed vipers were recorded to feed on birds (e.g., *Anthus campestris*) and mammals (e.g., *Rattus norvegicus* and *Apodemus mystacinus*) [16]. The venom of *M. l. lebetina* was found to be highly potent, with an LD_50_ value of 7.58 mg/kg, after intraperitoneal administration in mice [18]. The viper is the only medically important snake species in Cyprus [19], with more than fifteen annual snakebite cases recorded on average [20]. Human envenoming caused by venom of *M. l. lebetina* resulted in serious life-threating complications, such as swelling and edema at the bite site, and tissue necrosis following hypotension shock, hemorrhage, and melanoderma [21,22,23]. Approximately 2800 km away from the Cypriot *M. l. lebetina*, the subspecies *M. l. cernovi* occupies a unique habitat around the Kopet Dagh mountain range [24]. The viper is found in the diverse desert and montane-steppe biotopes and even occurs in the mountains up to 1500 m above sea level [25]. *Macrovipera l. cernovi* mainly feeds upon small birds (e.g., *Alectoris chukar* and *Galerida cristata*) and rodents (e.g., *Rhombomys opimus*) (personal observation), similar to its sister subspecies in Cyprus. With a massive body size and large venom glands, *M. l. cernovi* can release high volumes of bioactive venom. Human patients following snake bites by Iranian *M. l. cernovi* experienced significant systemic alterations such as a reduction in platelets and higher creatine kinase levels in serum among the local common symptoms [25].

Considering the previous studies of *M. lebetina* subspecies venom, the present study aimed to unravel the proteomic details of the venom of Iranian *M. lebetina cernovi* and Cypriot *M. lebetina lebetina*, and to enhance our knowledge of the geographical variability that is potentially present in the venom of *M. lebetina*. Hopefully, the results will provide in-depth insights into the diversity of *M. lebetina* venom, influencing the design of more effective immunizing mixtures for future antivenom production and clinical application in the region.

## 2. Results

### 2.1. An Overview of Proteomic Strategies for Profiling Venoms of M. l. cernovi and M. l. lebetina

**Figure 2:**
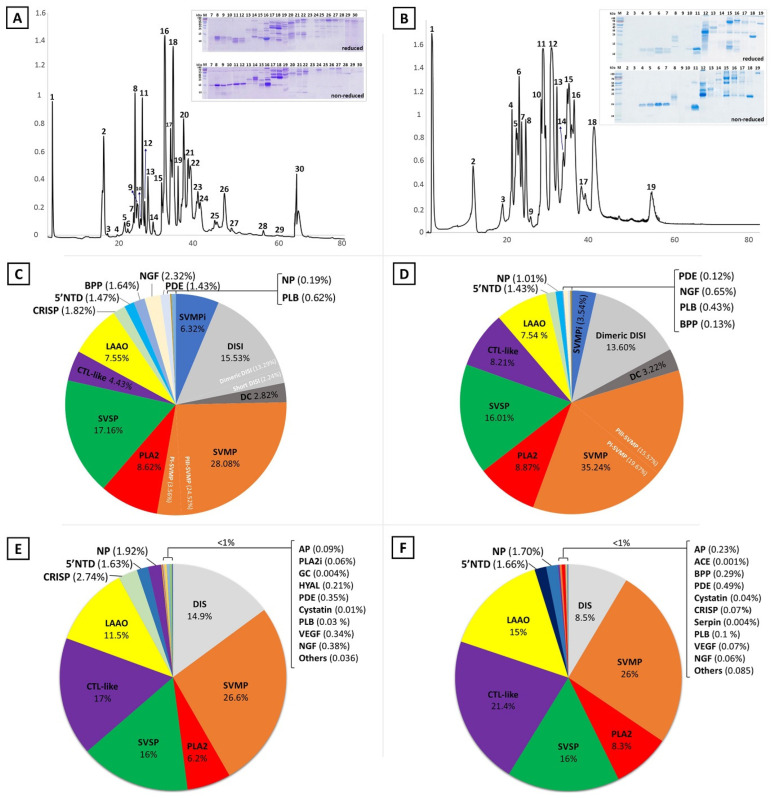
Venomics analysis of *M. l. lebetina* from Cyprus and *M. l. cernovi* from Iran. (**A**) Reverse-phase chromatographic separation of the venom proteome of *M. l. lebetina*, and (**B**) *M. l. cernovi*. The chromatographic fractions of both venoms were collected manually and analyzed by SDS-PAGE in both, reduced (upper section) and non-reduced (lower section), condition. (**C**,**E**) The pie charts indicate the identified protein families and their relative abundances (as percentage of total detected proteins) within the venom of *M. l. lebetina*, and (**D**,**F**) *M. l. cernovi* as determined either from the peak areas of reverse-phase chromatography with the help of SDS-PAGE protein bands (**C**,**D**) or based on the normalized spectral abundance factor from single-shot whole venom bottom-up proteomics (**E**,**F**). Acronyms: SVMP, snake venom Zn^2+^-metalloproteinase; PLA2, phospholipase A2; SVSP, snake venom serine protease; CTL-like, Snaclec, C-type lectin-like protein; LAAO, l-amino acid oxidases; DIS, disintegrin; CRISP, cysteine-rich secretory protein; NGF, nerve growth factor; VEGF, vascular endothelial growth factor; PDE, phosphodiesterase; AP, aminopeptidase; ACE, angiotensin-converting enzyme; BPP, bradykinin-potentiating peptides; PLB, phospholipase B; PLA2i, phospholipase A2 inhibitor; HYAL, hyaluronidase; NP, natriuretic peptide; 5′NTD, 5′-nucleotidase; DC, disintegrin-like/cysteine-rich fragment of PIII-SVMP; SVMPi, snake venom metalloproteinase inhibitor; NGF, nerve growth factor.

We applied different analytical approaches to comprehensively profile the venom proteomes of *M. l. cernovi* (Iran) and *M. l. lebetina* (Cyprus). We initially utilized the classical venomics method, starting with separating the venom proteome using reversed-phase ultrahigh-performance liquid chromatography (RP-UHPLC) and then manually collecting the detected chromatographic peaks. We next subjected the fractions to sodium dodecyl sulphate polyacrylamide gel electrophoresis (SDS-PAGE) analysis for further separation based on molecular weights under non-reduced and reduced conditions (Figure 2A,B and Figure S1, Supplementary Materials). The chromatographic fractions were identified by subsequent liquid chromatography tandem-mass spectrometry (LC-MS/MS) analyses of peptides obtained from in-gel tryptic digestion of SDS-PAGE protein bands. In addition, we applied intact mass measurement for the fractions that did not appear in SDS-PAGE. Since snake venoms contain a significant amount of low-abundant protein and low molecular weight peptide classes, they are mostly lost during the fraction collecting and in-gel digestion processes. In this case, for more profound identification, we also used single-shot proteomics, where the proteomes of whole venoms were subjected directly to in-solution digestion. We separated all resulting peptides online (RP-UHPLC) in this method and analyzed them via electrospray ionization high-resolution tandem mass spectrometry (ESI-HR-MS/MS).

The recorded data were searched against a Viperidae database, and peptides from each LC fraction, related gel bands, and whole venoms were identified independently. Supplementary Table S1 presents the features of molecular mass, MS/MS derived sequence, protein annotation, and quantification of each reverse-phase eluted fraction. HPLC peak-area calculations as a percentage of total venom proteins (for in-gel protein digestion) and the normalized spectral abundance factor (NSAF) in the case of shot-gun proteomics datasets were used for quantitative analysis. Panels C-F of Figure 2 show the composition profiles and relative abundances of protein families from the proteomes of both targeted venoms. 

### 2.2. The Venom Proteome of Cypriot M. l. lebetina

Figure 2A shows the RP-HPLC elution profile of female Cypriot *M. l. lebetina* venom, and the SDS-PAGE protein pattern of each collected fraction. The chromatogram revealed a high level of venom complexity, considering at least 30 elution peaks. LC-MS/MS analysis of peptides obtained from in-gel digestion and intact analysis of collected fractions identified 15 protein families (Figure 2C, and Table S1, Supplementary Materials). The most-abundant toxin groups included SVMP, SVSP, DISI, PLA2, CTL-like, disintegrin-like/cysteine-rich domain (DC), and L-amino acid oxidase (LAAO), together accounting for more than 80% of the venom’s protein content. SVMP (28.08%), DISI (15.53%) and DC (2.82%), with an abundance of ~46%, were the most expressed toxins in the venom. Among the SVMP toxin subfamilies, we detected PIII-SVMP (24.52%) and PI-SVMP (3.56%) subfamilies in the venom of *M. l. lebetina*. DISI as small polypeptides were also present in the venom, possibly produced by C-terminal domain proteolytical cleavage of PII-SVMP precursors or directly encoded as mRNA in the venom [27,28,29]. The presence of DC in the venom can also result from post-translational processing of PIII-SVMP in venom glands [30]. Other toxin families that accounted for more than 1% of the venom proteins included SVSP (17.16%), PLA2 (8.62%), LAAO (7.55%), snake venom metalloprotease inhibitor (SVMPi; 6.32%), CTL-like protein (4.43%), cysteine-rich secretory protein (CRISP; 1.82%), bradykinin-potentiating peptide (BPP; 1.64%), 5′-nucleotidase (5′NTD; 1.47%), nerve growth factor (NGF; 2.32%), natriuretic peptide (NP; 1.92%) and phosphodiesterase (PDE; 1.43%).

In addition, a single-shot data-dependent acquisition (DDA) experiment of whole digested venom yielded a higher number of identified peptides, resulting in the detection of many low abundant proteins (less than 1% abundance) among the others in the venom (Figure 2E). They included aminopeptidase (AP), hyaluronidase (HYAL), glutaminyl-peptide cyclotransferase (GC), cystatin, phospholipase B (PLB), vascular endothelial growth factor (VEGF), phospholipase A2 inhibitor (PLA2i), and also trace amounts of cellular proteins (others).

The combination of two proteomic approaches detected at least 18 protein families in the venom of Cypriot *M. l. lebetina*, and together provided greater insight into the venom components.

### 2.3. The Venom Proteome of Iranian M. l. cernovi

The reversed-phase LC-separated pattern of Iranian *M. l. cernovi* venom uncovered 19 chromatographic peaks (Figure 2B and Figure S1, Supplementary Materials). Proteomic analysis of collected fractions resulted in the qualification and quantification of 14 protein families (Figure 2D and Table S2, Supplementary Materials). The PI and PIII classes of snake venom Zn^2+^-dependent metalloproteinase (SVMP) represented 19.67% and 15.57% of the venom content, respectively, and together with proteolytic products of SVMP (DC; 3.22% and DISI; 13.60%) comprised the most abundant gene family expressed as proteins (52.06%) in the Iranian *M. l. cernovi* venom. Other high-abundant protein/peptide families were PLA2 (8.87%), SVSP (16.01), CTL-like protein (8.21%), LAAO (7.54%), 5′NTD (1.43%), SVMPi (3.54%) and NP (1.01%).

Furthermore, the whole digested venom was subjected to direct (shot-gun) LC-MS/MS analysis for further investigation of the composition and complexity of *M. l. cernovi* venom. The result discovered more than 18 protein families, and the method was sensitive to detect the families with less than 1% relative abundance, including AP, angiotensin-converting enzyme (ACE), BPP, PDE, Cyastatin, CRISP, Serpin, PLB, VEGF, NGF and others as illustrated in Figure 2F.

### 2.4. Comparison between Venom Proteomes of M. lebetina Subspecies

Reverse-phase chromatographic profiles of *M. l. cernovi* (Iran) and *M. l. lebetina* (Cyprus) venoms yielded 19 and 30 distinctive eluted peaks, respectively (Figure 2A,B). Gel electrophoretic patterns of collected fractions from the venoms also displayed that some chromatographic peaks contained multiple protein bands (Figure 2A,B and Figure S1, Supplementary Materials), which support the identification of different protein families from each fraction by LC-MS/MS (Tables S1 and S2, Supplementary Materials). Despite the various number of chromatographic peaks, we identified 15 and 14 protein/peptide classes in the venom of *M. l. lebetina* and *M. l. cernovi* using the classical venomics approach (Figure 2C,D). Furthermore, additional analysis with a shot-gun proteomic strategy resulted in 18 protein families besides the minor cellular proteins for both venoms (Figure 2E,F). The result revealed that the two viper venoms share similar protein classes. However, the relative occurrence of these toxins was different in each snake subspecies. They shared proteins belonging to SVMP, PLA2, SVSP, CTL-like, LAAO, CRISP, 5′NTD, BPP-NP, DISI, PDE, NGF, PLB, SVMPi, AP, VEGF, and Cystatin classes. On the contrary, the two venoms each contained trace amounts of unique components such as GC, PLA2i, and Hyal in *M. l. lebetina*, and Serpin in *M. l. cernovi*.

In Table 1, we detailed the proteome composition of *M. lebetina* subspecies venoms, investigating so far by different proteomics methodologies. Considerable differences are shown between the expressed toxins and their relative abundances in the venoms of *M. lebetina* subspecies. Although various methods can highly impact qualitative and quantitative outputs, the result demonstrated that the *M. lebetina* subspecies venoms are dominated by five major toxin families: SVMP, PLA2, SVSP, CTL-like, and DISI. Among all expressed toxins, SVMP comprises a significant part of *M. lebetina* venom proteomes, especially in the venom of *M. l. transmediterranea*, with 67%, roughly 2-fold more than the others. Interestingly, a significant difference was also detected among the P-classes of SVMP, revealing that P-I class toxins were more abundant than P-III ones in the venoms of *M. l. cernovi* and *M. l. obtusa* than in *M. l. lebetina* and *M. l. transmediterranea*, and vice versa. Apart from the toxin families abundantly expressed in the venoms of *M. l. cernovi* and *M. l. lebetina*, direct LC-MS/MS analysis of the peptide mixtures generated by in-solution digestion of the whole venoms revealed a considerable number of low-abundant protein classes (<1%). Notably, just a few of them (e.g., Hyal, PDE and VEGF) have been reported in other *M. lebetina* subspecies venoms until now. This probably is due to employing 1D and/or 2D gel electrophoresis/MS approaches for protein identification. As a result, low-abundant protein classes are excluded or less represented in gel patterns, and analysis of the yielded peptides from the digestion of gel spots cannot detect them without pre-gel enrichment.

## 3. Discussion

The proteomic analysis of venoms from *M. l. lebetina* and *M. l. cernovi* provides a snapshot of the toxin arsenal of *M. lebetina* members. The data presented here revealed that the protein family compositions of two *M. lebetina* subspecies are more complex than previously reported [31]. The number of identified proteins obtained here exceeded those documented by other *M. lebetina* subspecies [11,12,13], likely due to combining in-gel and in-solution digests of the venoms and fractions using a more sensitive mass spectrometer. Venom profiles between the analyzed *M. lebetina* subspecies indicate dominant expression of proteins belonging to SVMP, PLA2, SVSP, CTL-like, and DISI families. It is known that some major toxins can play a significant role in the pathophysiology of envenoming, for instance, disrupting the extracellular matrix of the vascular subendothelium (by SVMPs) [32], degradation of fibrinogen (by SVSPs) [33], inducing vascular hyperpermeability (by VGEF) [34], and promoting hypotension (by BPP/NP) [35]. However, the in vivo effects of most of the venom components are still poorly understood. Recently, clinical observations of human patients following Cypriot *M. l. lebetina* [19] and Iranian *M. l. cernovi* [25] envenoming have shown common local symptoms like edema and dermal necrosis, which seems mostly related to PLA2 and SVMPs toxins in both venoms [36]. Also, the clinical data of snakebite victims envenomated by *M. l. lebetina* revealed hypotension shock [19], possibly due to BPP/NP actions. In the case report of *M. l. cernovi* bites, the patients had significantly met higher creatine kinase (CK) levels [25]. An elevated serum CK level is a relatively common consequence of some viper envenomings, as a result of rhabdomyolysis or skeletal muscle damage, which is a useful marker of myotoxicity [37]. However, caution should be exercised when considering CK as a clinical indicator for early recognition of myotoxicity based on the observation of non-envenomed patients with early increased CK [38,39]. Clinical signs of *M. l. cernovi* snakebites furthermore evidenced that the venom induces thrombocytopenia [25], as a consequence of CTL-like toxin, described as the most relevant action of the toxin [40]. CTL-like protein generates platelet aggregation by indirectly modulating the von Willebrand factor (vWF) and promoting thrombocytopenia [41,42]. In addition, the impact of thrombocytopenia can contribute to the extent of systemic hemorrhage induced by SVMPs, as observed in the case of *Bothrops* species envenomings [40,43].

Overall, the toxin profile data delivers molecular evidence for understanding the clinical consequences following *M. lebetina* envenomings, such as local pain, edema, necrosis, and systemic manifestations, including hemorrhage, hypotension, coagulopathy and thrombocytopenia. Among all toxins, SVMP constitutes a significant portion of the total proteins in many Viperidae venoms [44,45], including *M. lebetina* venoms. Although SVMP is shown up in different subclasses (PI-, PII-, and PIII-SVMP), the biological function of each subclass can appear diverse from the other [46]. For example, venomics data of *M. lebetina* subspecies unveiled that the P-I class was less abundant than P-III in the venoms of *M. l. lebetina* and *M. l. transmediterranea* than *M. l. cernovi* and *M. l. obtusa*. While PIII-SVMPs induce hemorrhage by disrupting the extracellular matrix of the vascular endothelium, PI-SVMP provoke consumptive coagulopathy by fibrino(geno)lytic activities as well as persistent disruption of muscle tissue [47,48] but lacking from hemorrhagic activity [48,49,50,51,52]. Noteworthy, though myotoxicity was initially thought to be the result of PLA2 actions, there is now evidence that PI-SVMP are also explicitly involved in the disruption of muscle tissue [47]. In addition, proteomic data showed that PLA2 comprise a small part of the venom proteome (8–14%) of *M. lebetina* subspecies. Thus, we expect to see a more potent myonecrotic effect and minor systemic hemorrhage following envenoming by *M. l. obtusa* and *M. l. cernovi* compared to the two other ones. This is in agreement with the strong hemorrhagic activity of *M. l. transmediterranea* venom [53,54], and the clinical signs of potent myotoxicity in the venoms of *M. l. obtusa* and *M. l. cernovi* [16,25]. However, comprehensive knowledge about the action of venom toxins and their contribution to the clinical features of envenoming in humans has mostly remained unclear and needs to be investigated in future studies.

Several regional antivenoms have been produced using the venom of local *M. lebetina* subspecies during the hyperimmunization process, such as those of the Razi Vaccine & Serum Research Institute (polyvalent snake antivenom), the Institut Pasteur d’Algerie (anti-viperin), the Egyptian Organization for Biological Products & Vaccines (vacsera) and the Institut Pasteur du Tunis (gamma-vip) [55]. However, there is a lack of knowledge on the efficacy of these antivenoms against the regional used venoms and even other *M. lebetina* subspecies. A recent study showed that venoms of *M. lebetina* subspecies are highly potent in their ability to procoagulate, and their activation patterns of clotting factors are variable biochemically, influencing antivenom cross-neutralization efficacy [10]. Therefore, antivenoms produced without including the targeted venom are not entirely effective in treating the envenomed patients. Given that *M. l. cernovi*, *M. l. obtusa* and *M. razii* are distributed in Iran, it is unclear which *Macrovipera* venom species or subspecies were used for antivenom production by the producers [6]. In addition, in the case of *M. l. lebetina* envenomation in Cyprus, the Egyptian polyvalent antivenom (vacsera) is used for snakebite treatment (drawn from a personal conversation with pharmacies in Cyprus). However, it is unclear if *M. l. lebetina* venom was included in the antivenom production. It is appropriate to have polyvalent antivenoms targeting the toxins of all *Macrovipera* venoms instead of a single snake species or subspecies. This may be possible as reported in the case of Inoserp Europe polyvalent antivenom, which showed promising neutralizing potency against the venoms of *M. lebetina* subspecies and *M. schweizeri* [10,56].

The venoms of *M. l. cernovi*, *M. l. obtusa* and *M. l. lebetina* were found to contain significant quantities of endogenous tripeptide metalloprotease inhibitors (SVMPi). The tripeptides were also detected previously from venoms of different species, e.g., *Trimeresurus mucrosquamatus* [57], *Bothrops asper* [58], *Echis carinatus sochureki* [59], *Echis ocellatus*, *Cerastes cerastes cerastes* [60], *Deinagkistrodon acutus* [61], *Vipera ammodytes transcaucasiana* and *Vipera ammodytes montandoni* [62], and various species of rattlesnakes [63], confirming their role in reducing the proteolytic activity of SVMPs during storage in the venom gland to prevent self-intoxication [59]. They appear to be among the essential components of snake venoms, which contain significant amounts of SVMP toxins. However, their presence in the venom of other snakes requires intact-mass profiling due to their low-molecular weight (<500 Da), as utilizing gel electrophoresis for venom separation before MS analysis failed to detect them, for example, as seen in the case of *M. l. transmediterranea* venomics. SVMPi-related transcripts were detected from the venom gland transcriptomes of *Echis ocellatus*, *Cerastes cerastes cerastes* [60] and *Daboia russelii* [64] encoding tandem-repeated peptides, even containing C-type natriuretic peptide (CNP) and poly-His poly-Gly (pHpG) domains. Although these peptides are separately detectable from proteinaceous components in snake venoms, they are encoded from the same transcript and possibly liberated by posttranslational processes. It should be noted that the molecular evolutionary history of SVMPi has not yet been described. In addition to the most abundant proteins, many low-abundance proteins are also detected in the venoms of *M. l. cernovi* and *M. l. lebetina*. However, the actual contribution of most of them to the venom and envenoming is entirely uncertain and needs further research attention.

Considering the variety of structures and functions of snake venom toxins, it is expected that these substances are used as pharmacological tools and as prototypes in drug development [65,66]. Besides the severe clinical concerns marked in humans following *M. lebetina* envenoming, studies have shown the therapeutic potential of whole venoms and isolated toxins of *M. lebetina* subspecies, particularly the cytotoxic activities against several types of human tumor cells [67]. Nevertheless, the detailed mechanism behind the actions is still largely unknown and needs further research [67].

## 4. Conclusions

The present study revealed the qualitative and quantitative details of the venom proteomes of Iranian *M. l. cernovi* and Cypriot *M. l. lebetina*, employing RP-HPLC, SDS-PAGE, and high-resolution mass spectrometry. The finding showed that both venoms contain complex components which have not been reported previously. SVMP, PLA2, SVSP, CTL-like, LAAO, DISI, and SVMPi are the major protein/peptide classes presented in the venoms. By using the shot-gun proteomics strategy, we have also detected the proteins that had not been previously detected in the venom of *M. lebetina* subspecies, e.g., PLB, NGF, PDE, AP, ACE, Serpin, and Cystatin. This information may help to improve the therapeutic management of *M. lebetina* snakebite by predicting the clinical effects of the venoms. It also may influence the design of more effective immunizing mixtures for future antivenom production in the region. However, additional research using samples from different geographical localities is essential to resolve in-depth geographical variation in the venom composition of *M. lebetina* subspecies. In addition, further analysis is critical to characterize the *in-vivo* activities and biological roles of these venom components.

## 5. Materials and Methods

### 5.1. Venoms

The venom sample of *M. l. cernovi* was sourced from the two adult female specimens collected from Khorasan Razavi, Iran, whereas the *M. l. lebetina* sample was collected from the two adult female specimens (F2 generation of blunt-nosed vipers) originally caught nearby the cities of Paphos and Polis (Paphos distinct, Cyprus). The venoms of each subspecies were pooled, lyophilized immediately and stored at −20 °C until used for further analysis. The samples were provided by SMK and SJ under permission numbers of 40,401,008 (issued by the Department of the Environment of Iran) and 02.15.001.003, 04.05.2002.005.006 (issued by the Department of Environment of Cyprus), respectively.

### 5.2. Chemicals

All chemicals were purchased from Sigma-Aldrich (Sigma-Aldrich, MO, USA), and VWR chemicals (VWR international, Darmstadt, Germany). RapiGest SF surfactant was purchased from Waters (Waters Corporation, MA, USA). The mass spec grade Trypsin/Lys-C mix was purchased from Promega (Promega, Mannheim, Germany). Zip Tip C18 was purchased from Millipore (Bedford, MA, USA).

### 5.3. Protein Concentration Estimation

The protein concentrations were determined before each proteomics analysis using a standard Bradford assay (Biorad, Hercules, CA, USA), with bovine serum albumin (BSA) as a reference. Absorbance was measured spectrophotometrically at 595 nm on a BioTek Synergy 2 plate reader (BioTek, Winooski, VT, USA) with Gen5 software (version 2.01).

### 5.4. HPLC and SDS-PAGE Separation

Five milligrams of crude venoms were dissolved in 500 µL of 5% acetonitrile/water (ACN/H_2_O) containing 0.1% trifluoroacetic acid (TFA) and were centrifuged at 12,000× *g* at 4 °C for 5 min (Beckman Coulter, Krefeld, Germany) to remove debris. Fifty micrograms of proteins from each sample were separated using a bioZen™ LC Column (3.6 µm, 50 × 2.1 mm, pore size of 200 Å; Intact XB-C8, Phenomenex, CA, USA) connected to a Dionex UltiMate 3000 RSLC HPLC system (Thermo Fisher Scientific, Waltham, MA, USA). The chromatographic analyses were performed at a flow rate of 800 µL/min using H_2_O with 0.1% trifluoroacetic acid (TFA) as mobile phase A and ACN with 0.1% TFA as mobile phase B. The following gradient elution profile was applied as follows: isocratic (5% B) for 5 min, followed by 5–45% B over 70 min, 40–70% B over 20 min, and re-equilibration (5% B). The absorbance of fractions was measured at wavelengths of 215 nm, and the fractions were collected manually. The collected fractions were vacuum dried and further applied to non-reducing and reducing SDS-PAGE, using 15% acrylamide gels with a pre-stained protein ladder (Thermo Fisher Scientific, Dreieich, Germany). The gels were stained with Coomassie Brilliant Blue G-250 (Merck, Darmstadt, Germany), and the protein bands were excised and subjected to in-gel tryptic digestion. The scanned gel picture was analyzed for the relative density of protein spots using ImageJ software (https://imagej.nih.gov/ij/list.html (accessed on 1 March 2022)).

### 5.5. LC-MS/MS Analysis

Equal amounts of the whole venoms were diluted with 50 mM ammonium bicarbonate buffer, containing 0.1% RapiGest, and incubated for 15 min in a thermomixer at 80 °C (Eppendorf Thermomixer C, Hamburg, Germany) to complete proteome solubilization. The venom and SDS-PAGE band samples were reduced and alkylated with 100 mM dithiothreitol (DTT) at 56 °C for 15 min, and 200 mM iodoacetamide (IAA) at room temperature (dark place) for 30 min, respectively. The digestion was performed with mass spectrometry grade Trypsin/Lys-C mix (1:25 enzyme to proteins ratio) at 37 °C. The reaction was stopped after 16 h by adding formic acid to 1.5% and incubating at 37 °C for 10 min prior to centrifugation. The peptide samples were desalted before the LC-MS/MS measurement by using ZipTip C18 and then concentrated using Eppendorf Concentrator Plus (Eppendorf, Hamburg, Germany). The resulting peptides were separated using a Dionex UltiMate 3000 RSLC UHPLC system (Thermo Fisher Scientific) on a Kinetex C18 (2.1 mm × 100 mm, 2.6 µm 100 Å particle size) column (Phenomenex, CA, USA) coupled to a Q Exactive HF-X benchtop Orbitrap mass spectrometer (Thermo Scientific, Bremen, Germany) by heated electrospray ionization (HESI-II) ion source (Thermo Fisher Scientific). For each sample, three technical replicates were performed. The chromatographic analysis was performed at 250 µL/min flow rate with water/0.1% formic acid (mobile phase A) and acetonitrile/0.1% formic acid (mobile phase B). The gradient elution of 120 min was applied as follows: isocratically (2% B) for 5 min, followed by 2–40% B over 100 min, 40–50% B over 5 min, 50–98% B over 2 min, and re-equilibration in 2% B. The mass spectrometer was operated in data-dependent acquisition mode (top-15 DDA) with the following parameters in full MS scans: mass range of *m*/*z* 350 to 1650, mass resolution of 120,000, automatic gain control (AGC) target of 3 × 10^6^, injection time (IT) of 50 ms, and MS/MS scans with mass resolution of 30,000, AGC target of 1 × 10^5^, IT of 55 ms, isolation window Δ(*m/z*) 1.3, normalized collision energy (NCE) of 28, and dynamic exclusion of 30 s.

Top-down mass measurements were applied to the fractions not revealed in the SDS-PAGE as follows: fractions were loaded onto a Jupiter C18 (4.6 mm × 250 mm, 3 µm 300 Å particle size) column (Phenomenex, Torrance, CA, USA) independently. A Dionex UltiMate 3000 RSLC UHPLC system (Thermo Fisher Scientific, Bremen, Germany) was interfaced with a Q Exactive HF-X benchtop Orbitrap mass spectrometer (Thermo Fisher Scientific, Bremen, Germany) using HESI-II ion source (Thermo Fisher Scientific). Chromatographic analysis was performed at a flow rate of 400 µL/min, using water/0.1% FA (mobile phase A) and ACN/0.1% FA (mobile phase B). The gradient of 90 min was applied as follows: isocratically (2% B) for 5 min, 2–55% B over 85 min, and re-equilibration in 2% B. The mass spectrometer was operated in data-dependent acquisition (top-5 DDA) with the following parameters in full MS scans: mass range *m*/*z* 350–1650, mass resolution of 120,000 (@ *m*/*z* 200), AGC target of 1 × 10^6^, IT of 100 ms and MS/MS scans: mass resolution 30,000 (@ *m*/*z* 200), AGC target of 1 × 10^5^, IT of 120 ms, isolation window Δ(*m/z*) 1.3, dynamic exclusion 30 s, and normalized collision energy (NCE) of 30.

### 5.6. Data Analysis

The raw files were searched against the UniProt protein database that was taxonomically set to the Viperidae (taxon ID # 8689) databases (downloaded on 1 March 2021) using Proteome Discoverer (PD) software suite, version 2.4 (Thermo Fisher Scientific) for bottom-up proteomics, and TopPIC Suite, version 2.0 [68]. For PD, two search engines, SEQUEST and MS Amanda, were used with the peptide precursor and fragment ion mass tolerance set to 10 and 0.5 ppm, respectively. The parameters were assigned to a maximum of two missed cleavage sites of trypsin digestion and a minimum peptide length of 6. The dynamic modification was set to oxidation (+15.995 Da [M]) and static modification to carbamidomethyl (+57.021 Da [C]). Percolator node was used to validate identified peptide-spectrum matches (PSMs) and filter the data with parameters of a strict target FDR (false discovery rate) of 0.01 and a relaxed target FDR of 0.05. The MaxQuant contaminant database was used to mark contaminants in the results file, and high confidence proteins in the master group with at least two unique peptides were considered. In TopPIC software, the signal-to-noise ratio was set to 2, the precursor window size set to Δ*m*/*z* = 4, mass error tolerance was set to 10 ppm, and the decoy database was used to filter the spectrum with FDR cut-off of 0.01. For shot-gun proteomics, the relative quantitation of identified proteins from the PD output was calculated based on the normalized spectral abundance factor (NSAF) [69,70] manually.

## Figures and Tables

**Table 1:** Comparison of toxin families detected in the venom proteomes of *M. lebetina* subspecies, investigated by almost the same proteomic technique. Overview of the relative abundances of toxin families in the venom of Cypriot *M. l. lebetina* and Iranian *M. l. cernovi* along with published data on Russian *M. l. obtusa* [13], Armenian *M. l. obtusa* [12] and Tunisian *M. l. transmediterranea* [11].

Protein Family	% Of Total Venom Proteins				
	*M. l. lebetina* (Cyprus)	*M. l. cernovi* (Iran)	*M. l. obtusa* (Armenia)	*M. l. obtusa* (Dagestan)	*M. l. transmediterranea* (Tunisia)
P-I snake venom Zn^2+^-metalloproteinase	3.56	19.67	27.8	14.6	-
P-III snake venom Zn^2+^-metalloproteinase	24.52	15.57	4.3	9.4	67
Dimeric disintegrin	13.29	13.6	8.5	5.5	6
Medium disintegrin	-	-	-	<1	-
Short disintegrin	2.24	-	2.8	7.4	<1
Disintegrin/cysteine-rich fragment	2.82	3.22	1.7	0.6	1
Serine proteinase	17.16	16	14.9	23.4	9
Phospholipase A2	8.62	8.87	14.6	13.6	4
C-type lectin-like	4.43	8.21	14.8	8.7	10
L-amino acid oxidase	7.55	7.54	1.7	2	-
Cysteine-rich secretory protein	1.82	<1	2.6	1.1	-
Bradykinin-potentiating peptides/natriuretic peptide	1.83	1.14	5.3	5.6	<1
Hyaluronidase	<1	-	-	<1	-
Phosphodiesterase	1.43	<1	-	<1	-
5′-nucleotidase	1.47	1.43	-	<1	-
Snake venom Zn^2+^-metalloproteinase inhibitor	6.32	3.54	-	4.8	-
Phospholipase B	-	<1	-	-	-
Nerve growth factor	2.32	<1	-	-	-
Vascular endothelial growth factor	<1	<1	-	-	2

## Data Availability

The bottom-up proteomic data presented in this study are available in ProteomeXchange Consortium (http://proteomecentral.proteomexchange.org (accessed on 1 September 2022)) via the PRIDE partner repository with the dataset identifier PXD036466.

## Supplementary Materials

The following materials are available online at https://www.mdpi.com/article/10.3390/toxins14100716/s1, Tables S1 and S2: List of identified proteins and peptides from *M. l. cernovi* and *M. l. lebetina* venoms. Figure S1: RP-HPLC and SDS-PAGE separation of the venom proteome of *M. l. lebetina*, and *M. l. cernovi*.
